# The evolution of radiographers interpreting radiographs in Australia and New Zealand: Nomenclature matters

**DOI:** 10.1002/jmrs.816

**Published:** 2024-08-26

**Authors:** Michael J. Neep, Andrew Murphy

**Affiliations:** ^1^ Department of Medical Imaging Logan Hospital Meadowbrook Queensland Australia; ^2^ School of Clinical Sciences Queensland University of Technology Brisbane Queensland Australia; ^3^ Medical Imaging and Nuclear Medicine Queensland Children's Hospital, Children's Health Queensland Hospital and Health Service South Brisbane Queensland Australia

## Abstract

This editorial summarises the evolution and positive impact that radiographer preliminary image evaluation has on patient care. It also highlights the importance of using consistent and clear terminology when referring to when radiographers alert significant pathology to the referring clinical team and radiologists.

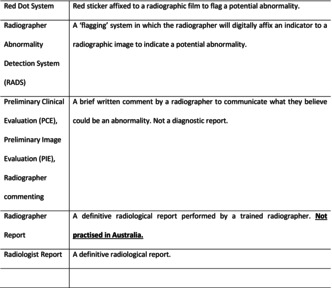

Diagnostic errors in medical imaging can have major implications on patient outcomes. Unfortunately, radiology reports are frequently not available to inform patient management or to enable faster discharge. The delay in the availability of the radiology report can contribute to missed, incomplete or incorrect diagnoses, which can lead to suboptimal patient treatment. There is growing consensus among medical imaging professionals internationally that these risks to patient care can be mitigated by the radiographer alerting significant findings at the time of imaging.

The literature has documented that radiographers have been alerting significant findings for over 40 years. The initial evaluation of plain radiographic images for potential abnormalities by radiographers has been accepted practice in the United Kingdom since the early 1980s.^1^ In an attempt to reduce diagnostic errors in the emergency department, Berman et al proposed a system by which radiographers affixed a red sticker to plain X‐ray films they believed to be abnormal. The red sticker acted as a visual cue, alerting the referrer of a potential abnormality. It was not until the 1990s that red dot abnormality signalling systems were first introduced into Australia. Red dot abnormality signalling systems were often, and justifiably, criticised for the lack of transparency. The absence of documentation as to what the radiographer was flagging was problematic and sometimes dangerous, it was a communication flaw that left referrers and radiologists frustrated. To address this limitation, red dot abnormality signalling systems in the United Kingdom evolved in the 2000s to include a brief comment accompanying an examination, describing any abnormality flagged by the red dot.^2^ The brief accompanying interpretation, known initially as the ‘radiographer comment’, was officially termed the Preliminary Clinical Evaluation (PCE) in the United Kingdom in 2012.^2^ The decision to change the nomenclature from‘radiographer comment’ to ‘Preliminary Clinical Evaluation’ was made to clearly establish the difference between initial (preliminary) image interpretation (PCE) and clinical reporting by radiographers (radiographer reporting). Radiographer reporting in the United Kingdom involves appropriately trained radiographers that perform independent diagnostic reporting, the same as a radiologist.^2^ The 2000s also saw the first implementation of ‘radiographer commenting’ in Australia.^3^ Similarly to the United Kingdom, misunderstandings around the terminology being confused with diagnostic reporting resulted in a decision in 2017 to refer to ‘radiographer commenting’ as Preliminary Image Evaluation (PIE) in Australia.^4^ A PIE is a brief written description that acts in the same way as a ‘radiographer comment’ or PCE, in that it clearly communicates significant clinical findings to the referring clinician in the absence of a definitive radiologist report. The decision to steer away from using the term ‘radiographer comment’ was to help distinguish a clear difference between ‘radiographer commenting’ and ‘radiographer reporting’ similar to what was experienced in the United Kingdom. A systematic review published in JMRS in 2019^5^ included a table that provided clear definitions of the commonly used terms that are used to describe radiographer image interpretation (see Table 1). Both authors of this editorial are strong advocates of using the term PIE, however prior to the establishment of the more contemporary name PIE, both authors witnessed on multiple occasions health professionals and academics interchanging ‘radiographer comment’ with ‘radiographer reporting’. Interestingly, the findings of this 2019 systematic review found this heterogeneity in terminology to be prevalent in academic publications. Over the past 20 years, studies on Australian radiographers' ability to evaluate radiographs has shown significant variability in performance, partly due to inconsistent terminology, especially between ‘reporting’ and ‘commenting’. This inconsistency has caused confusion and concern among radiologists about the radiographers' roles. Clear, universal definitions are crucial to advance radiographers' roles in image interpretation and address interprofessional barriers. Although local centres may have different names for PIE, the authors strongly encourage all Australian radiographers, researchers and authors to be consistent with referring to when a radiographer is alerting significant pathology at the time of imaging as a Preliminary Image Evaluation (aka a PIE). This is to ensure that we are being clear and consistent with our terminology and subsequently to help promote the widespread implementation of radiographer PIE systems.

**Table 1:** Radiographic interpretation definitions in the literature.

Red Dot System	Red sticker affixed to a radiographic film to flag a potential abnormality
Radiographer Abnormality Detection System (RADS)	A ‘flagging’ system in which the radiographer will digitally affix an indicator to a radiographic image to indicate a potential abnormality
Preliminary Clinical Evaluation (PCE), Preliminary Image Evaluation (PIE), Radiographer commenting	A brief written comment by a radiographer to communicate what they believe could be an abnormality. Not a diagnostic report
Radiographer Report	A definitive radiological report performed by a trained radiographer. *Not practised in Australia*
Radiologist Report	A definitive radiological report

The authors of this editorial strongly believe that prior to the use of the term PIE, inconsistencies in interchanging terms such as ‘radiographer comment’ and ‘radiographer reporting’ contributed to the Royal Australian and New Zealand College of Radiologists (RANZCR) opposition of the use of radiographer interpretation systems in Australia.^6^ If RANZCR had all the facts, they would understand that PIE is a preliminary assessment at the time of imaging which is within the scope of the Australian radiographer. It is therefore highly likely, that RANZCR would understand that the radiographer flagging a misplaced nasogastric tube is more about patient safety than anything else—but when terminology becomes cloudy, it is hard not to sound the alarm.

The authors of this editorial are hopeful that this interprofessional barrier could be overcome with a clear and consistent use of the term PIE. PIE removes any connotations to reporting and is consistently defined in the Australian literature. Some centres may employ a variation of PIE that involves signalling ‘no abnormality detected’ or ascribe to a list of key pathologies – that does not change that the definition of PIE is consistently known as a preliminary evaluation and not a substitute for a radiology report.

Looking beyond the naming evolution of this patient safety mechanism, it is great to read in this edition of JMRS that PIE is being practiced in New Zealand.^7^ To date, studies investigating PIE within New Zealand have not been widely published. This study contributes to the existing evidence base that radiographers can make a positive impact on reducing missed pathologies in the emergency department. Lewis et al study^7^ indicated that following targeted x‐ray interpretation education, radiographers who participated in this six‐month study retained a high level of diagnostic accuracy when communicating their PIEs. These findings highlight the importance of continuous x‐ray interpretation training so to maintain this desired skill. Furthermore, regular auditing of x‐ray interpretation performance is valuable to monitor the accuracy of PIE interpretations and to highlight training needs of areas that are difficult to interpret.

With the ever‐growing evidence base, strengthened by the outcome of this New Zealand study,^7^ it is easy to acknowledge the value of a radiographer PIE that can complement a referring clinician's interpretation when a radiologist report is unavailable. PIE is a patient safety mechanism that ensures patients needs are met within an appropriate time frame. Radiographers in Australia are highly skilled, and have the desired skill set to comprehend what a significant finding is. It is also important to acknowledge the value of a radiographer documenting that an examination does not contain an abnormality. This communication to the referrer can enable a faster discharge. In the contemporary landscape of healthcare, where nurse practitioners and physiotherapists can request and interpret radiographs, radiographers can and do make a positive impact. There should be no conceivable scenario where a radiographer observed an urgent finding and ignores it, especially in Australia and New Zealand where radiographers are taught image interpretation in their undergraduate education. Just as the Medical Radiation Practice Board of Australia's policy titled Communicating safely—if urgent or unexpected findings are identified; if you see something, say something.^8^

## Conflict of Interest

The authors declare no conflict of interest.

## Data Availability

Data sharing is not applicable to this article as no new data were created or analyzed in this study.
